# Comparison of Various Anthropometric and Body Fat Indices in Identifying Cardiometabolic Disturbances in Chinese Men and Women

**DOI:** 10.1371/journal.pone.0070893

**Published:** 2013-08-12

**Authors:** Zhe-qing Zhang, Juan Deng, Li-ping He, Wen-hua Ling, Yi-xiang Su, Yu-ming Chen

**Affiliations:** 1 Guangdong Provincial Key Laboratory of Food, Nutrition and Health; School of Public Health, Sun Yat-sen University, Guangzhou, People's Republic of China; 2 Guangzhou Panyu Central Hospital, Guangzhou, People's Republic of China; INSERM/UMR 1048, France

## Abstract

**Background:**

Although many adiposity indices may be used to predict obesity-related health risks, uncertainty remains over which of them performs best.

**Objective:**

This study compared the predictive capability of direct and indirect adiposity measures in identifying people at higher risk of metabolic abnormalities.

**Methods:**

This population-based cross-sectional study recruited 2780 women and 1160 men. Body weight and height, waist circumference (WC), and hip circumference (HC) were measured and body mass index (BMI), waist-to-hip ratio (WHR), and waist-to-height ratio (WHtR) were calculated. Body fat (and percentage of fat) over the whole body and the trunk were determined by bioelectrical impedance analysis (BIA). Blood pressure, fasting lipid profiles, and glucose and urine acid levels were assessed.

**Results:**

In women, the ROC and the multivariate logistic regression analyses both showed that WHtR consistently had the best performance in identifying hypertension, dyslipidemia, hyperuricemia, diabetes/IFG, and metabolic syndrome (MetS). In men, the ROC analysis showed that WHtR was the best predictor of hypertension, WHtR and WC were equally good predictors of dyslipidemia and MetS, and WHtR was the second-best predictor of hyperuricemia and diabetes/IFG. The multivariate logistic regression also found WHtR to be superior in discriminating between MetS, diabetes/IFG, and dyslipidemia while BMI performed better in predicting hypertension and hyperuricemia in men. The BIA-derived indices were the second-worst predictors for all of the endpoints, and HC was the worst.

**Conclusion:**

WHtR was the best predictor of various metabolic abnormalities. BMI may be used as an alternative measure of obesity for identifying hypertension in both sexes.

## Introduction

Obesity can promote a cascade of secondary cardiometabolic pathologies such as hypertension, hyperlipidemia, insulin resistance, and hyperuricemia, alone or in combination, all of which exacerbate the progression of cardiovascular disease (CVD) [1]. The close association between either absolute total fat or adipose tissue distribution and these metabolic abnormalities has been well documented [2]. Nevertheless, controversy remains over which anthropometric parameter best defines obesity and conveys the highest risk of cardiometabolic disturbance. In recent years, waist-to-height ratio (WHtR) has been regarded as the best screening tool for detecting cardiometabolic risk factors, especially in Asians [3], [4], [5], [6]. Some studies have proposed the use of waist circumference (WC) or waist-to-hip ratio (WHR) [7], [8], [9], whereas others advocate their combined use [10], [11].

Although body mass index (BMI), WHtR, WC, and WHR are simple and convenient measures for epidemiological studies, their validity in measuring adiposity has been questioned because they do not directly measure the amount of adipose tissue and cannot differentiate between fat and lean mass [12]. Although it has been suggested that a more direct and accurate assessment of adiposity would have higher value in predicting obesity-related health risks, previous studies have produced inconsistent findings. Some studies have found that direct indictors exhibited better predictive performance than simple anthropometric parameters [13], [14], and others have found them to be equivalent [15], [16]. However, many studies have observed the discriminatory capability of those simpler measures to be more robust than the measures derived from dual-energy x-ray absorptiometry (DXA) [17], [18], [19], computed tomography (CT) [20], or bioelectrical impedance analysis (BIA) [11], [21]. Compared with more sophisticated methods such as magnetic resonance imaging, CT, and DXA, BIA is generally easy to use, portable, and much more affordable. It also poses no risk to patients [22]. A validation study with a Chinese sample demonstrated good agreement between BIA, MRI, and DXA [23]. However, it remains unclear whether the discriminative power of BIA-derived body composition measurements is superior to that of simpler adiposity indictors in identifying the risk of cardiometabolic abnormalities among Chinese.

This community-based cross-sectional study aimed to identify the best single predictor of cardiometabolic disturbance by comparing the ability of various anthropometric measurements (BMI, WC, WHtR, WHR) and direct body fat measurements [total body fat (BF), percentage body fat (%BF), trunk fat mass (TF), percentage trunk fat (%TF)] to discern who was at a higher risk of hyperlipidemia, hyperuricemia, hyperglycemia, or metabolic syndrome (MetS) among middle-aged Chinese women and men.

## Participants and Methods

### Study population

Two thousands seven hundred and eighty women aged 37–74 and 1160 men aged 49–94 were recruited by sending invitation letters to residential buildings, posting local advertisements, giving health talks, and through referrals from local communities in urban Guangzhou, Guangdong Province, China between October 2005 and June 2010. The recruitment procedure is described in more detail in [24]. We excluded participants with previously confirmed severe medical diseases such as cancer, stroke, and heart failure, along with those who were using medication known to affect lipid metabolism. All of the participants gave written informed consent and the study was approved by the Ethics Committee of School of Public Health of Sun Yat-sen University.

### Data collection

Staff members with relevant medical knowledge screened the potential participants using a telephone survey. Eligible participants were invited to local health centers or the School of Public Health at Sun Yat-sen University. Face-to-face interviews were conducted using structured questionnaires to collect participants' socio-demographic information, their own and their families' medical histories, medication information, smoking status, and alcohol consumption. Anthropometric measurements, physical examinations, BIA-body fat, and blood pressure measurements were conducted at the same time. A 24-hour physical activity questionnaire containing 19 items was used to estimate daily physical activity [24]. And the physical activity was calculated by the combination of the metabolic equivalent score (MET, kcal.kg^−1^.h^−1^) for each type of physical activity after multiplied by its duration per day (h/d) [25]. Venous blood was collected after overnight fasting (≥10 h), and serum was separated within 2 hours (at 4°C) and stored at −80°C before the analysis.

### Anthropometric measurements

Anthropometric indices were measured with participants dressed in light clothing and barefoot. BMI was calculated as weight (in kilograms)/height (in meters) squared. Waist and hip circumferences were measured at the level of the umbilicus, and at the level of the maximum girth between the iliac crest and the crotch, respectively. Each parameter was measured twice and a third measurement was carried out if the difference ≥2 cm. WHR was calculated as the ratio of the waist-to-hip circumferences. WHtR was calculated by dividing waist circumference by height. The absolute and the percentage of fat over the whole body and trunk were assessed by a bioelectrical impedance analysis (BIA) system using a Tanita TBF-418B (Tanita Corporation, Tokyo, Japan). The in-vivo reproducibility of the BIA system was 1.54%, 1.85%, 2.50%, and 2.79%, for the %BF, FM, %TF, and TFM, respectively.

### Blood pressure measurement

Two blood pressure measurements were performed at an interval of at least 10 minutes by a mercury sphygmomanometer while the participants were in a sitting position after ≥10 min. of rest. The average of the two values was used unless the deviation of the two measurements for systolic pressure was ≥4 mmHg or diastolic pressure ≥3 mmHg, in which case a third measurement was required.

### Serum analysis

Serum total cholesterol (TC) and triglycerides (TG) were measured by the enzymatic colorimetric method (Human, Wiesbaden, Germany). Direct methods were applied to assess HDL cholesterol (HDLc) and LDL cholesterol (LDLc) (Seikisui, Tokyo, Japan). The glucose oxidase method was used to detect glucose (Glu) (Homa, Beijing, China), and serum urine acid (UA) was measured by the enzymatic colorimetric method (Fenghui, Shanghai, China). All of these measures were performed by a Hitachi 7600–010 automatic analyzer (Hitachi, Tokyo, Japan). The variation coefficient for the serum measurements was 2.17% (at 5.03 mmol/L TC), 2.86% (at 1.14 mmol/L TG), 3.47% (at 1.70 mmol/L HDLc), 4.67% (at 2.65 mmol/L LDLc), 2.52% (at 4.45 mmol/L Glu), and 5.46% (at 303.5 mmol/L UA).

### Definition of cardiometabolic abnormalities

Hypertension was defined as the current use of antihypertensive medication, a systolic blood pressure of ≥140 mm Hg, or diastolic blood pressure of ≥90 mm Hg [26]. Dyslipidemia was defined according to the NCEP ATP III criteria [27]. Accordingly, participants with one or more of the following results were considered dyslipidemic: plasma cholesterol (TC) ≥6.22 mmol/L (240 mg/dl), triglycerides (TG) ≥2.26 mmol/L (200 mg/dl), LDLc ≥4.14 mmol/L (160 mg/dl), or HDLc <1.03 mmol/L (40 mg/dl). Hyperuricemia was diagnosed by serum uric acid ≥420 μmol/l (7.0 mg/dl) in men, or ≥350 μmol/l (6.0 mg/dl) in women [28]. Diabetes was defined as a fasting plasma glucose concentration ≥7.0 mmol/l (126 mg/dL) or treatment for diabetes, and impaired fasting glucose (IFG) was defined as a plasma glucose concentration between 5.6 and 6.9 mmol/l (100–125 mg/dl) [29].

Following the IDF criteria, metabolic syndrome was defined by central obesity (defined as waist circumference ≥90 cm in men or ≥80 cm in women, or a BMI >30 kg/m), plus any two of the following four factors: (1) TG concentration ≥1.7 mmol/L (150 mg/dL); (2) HDLc concentration <1.03 mmol/L (40 mg/dL) in men or <1.29 mmol/L (50 mg/dL) in women; (3) systolic blood pressure ≥130 or diastolic blood pressure ≥85 mm Hg or treatment for previously diagnosed hypertension; or (4) a fasting glucose concentration ≥5.6 mmol/L (100 mg/dL) or treatment for diabetes [30]. Given that the objective of this study was to establish the optimal measurements for the evaluation of MetS, the obesity requirement for this definition was omitted.

### Statistical analysis

The data were analyzed separately by sex. We examined the distribution of adiposity measures and other characteristics using the Kolmogorov-Smirnov test in Table 1. The results of continuous variables were presented as “mean ± standard deviation (SD)” when they followed a normal distribution, or mean (interquartile range) when they asymmetrically distributed. Categorical data were expressed as frequencies. The area under the receiver's operating characteristic curve (AUC) and the 95% confidence intervals (CIs) were used to compare the predictive ability of adiposity measurements of metabolic abnormalities. The AUC is a measure of the degree of separation between case and control subjects. The obesity measurements were separately categorized into four quartiles. The odds ratios (OR) and their 95% CIs for the presence of cardio-metabolic disturbances, compared using the highest to the lowest quartile of each obesity index, were estimated by logistic regression using the “enter” procedure with adjustments for age, physical activity, smoking, and alcohol status. All of the analyses were carried out using the SPSS statistical package (version 13.0, SPSS Inc, Chicago, IL), and P-values less than 0.05 (two-tailed) were considered to be significant.

**Table 1:** Characteristics of the participants*.

	Female		Male		
		N	Mean	SD	N
Age, year	55.0 (7.0)	2780	57.0 (8.0)		1160
Smokers, %	0.8	2780	52.2		1160
Alcohol drinker, %	1.9	2780	13.9		1160
Physical activity, MET·h/d#	29.7±5.9	2780	30.0±6.6		1160
Anthropometric measures					
BMI, kg/m^2^	23.1(4.1)	2756	23.7(3.9)		1155
Waist circumference, cm	80.8(12.2)	2779	85.9(10.5)		1159
Hip circumference, cm	93.4(8.0)	2779	94.2(7.0)		1157
Waist to hip ratio	0.86(0.09)	2779	0.91±0.06		1157
Waist to height ratio	0.52(0.08)	2779	0.52±0.05		1159
Body fat, kg	17.8(8.2)	1674	13.5(6.4)		683
Percentage body fat, %	30.9(8.2)	1674	19.8(6.5)		683
Trunk fat, kg	9.5(4.9)	1674	7.5(4.0)		683
Percentage trunk fat, %	29.5(10.2)	1674	20.4(8.6)		683
Biochemical indicators					
Total cholesterol (mmol/L)	5.51 (1.32)	2780	5.09 (1.25)		1160
LDLc (mmol/L)	3.60 (1.18)	2780	3.41 (1.10)		1160
HDLc (mmol/L)	1.45 (0.47)	2780	1.21 (0.38)		1160
Triglycerides (mmol/L)	1.30 (0.97)	2780	1.43 (1.16)		1160
Uric acid (mmol/L)	255 (194)	2464	330 (107)		1007
Fasting glucose (mmol/L)	4.60 (0.80)	2780	4.80 (0.90)		1160
Blood pressure					
Systolic (mmHg)	120 (22)	2778	124 (24)		1159
Diastolic (mmHg)	80 (14)	2778	80 (13)		1159

* : Continuous variables were presented as “mean ± standard deviation (SD)” when they followed a normal distribution, or mean (interquartile range) when they asymmetrically distributed.

# : Physical activity was evaluated by metabolic equivalent (MET) hours per day.

## Results

Table 1 summarizes the baseline characteristics. Men tended to have higher BMI, WC, and HC but lower absolute and percentage fat mass than women. According to the BMI criteria, 25.3% of women and 31.8% of men were considered to be either overweight or obese. Men had a significantly higher prevalence of hypertension (36.6% vs. 29.3%), hyperuricemia (13.4% vs. 6.7%), diabetes/IFG (15.5% vs. 12.1%), dyslipidemia (51.1% vs. 47.6%), and metabolic syndrome (37.9% vs. 33.6%) than women (all P<0.05).

According to the respective ROC curves, WHtR had the highest AUC values, and thus was the best predictor of all cardiometabolic disturbances in women, followed by WC, WHR, BMI, and HC. In men, no single index had a consistently higher AUC value than the others. However, WHtR demonstrated better discrimination for hypertension (0.67), and WHtR and WC had the equal-highest predictive power for dyslipidemia (0.64) and MetS (0.69). WHtR was also the second-best predictor of hyperuricemia (0.64 vs. 0.66 by BMI) and diabetes/IFG (0.60 vs. 0.62 by WHR). The BIA adiposity indices had substantially lower AUCs than the anthropometric indices (Table 2). The optimal cut-off value, sensitivity, specificity, positive and negative predictive values for the best obesity index in ROC analysis for predicting those metabolic risks were exhibited in Table S1. Figure S1 and S2 show the ROC curves of the best obesity measurement for predicting metabolic risks.

**Table 2:** The area under the curves of each adiposity variable for the presence of hypertension, dyslipidaemia, hyperuricemia, and metabolic syndrome in both genders.

	Hypertension	Dyslipidaemia	Hyperuricemia	Diabates/IFG	MetS
***Female***					
BMI	**0.66 (0.63,0.68)**	0.59 (0.57,0.61)	0.69 (0.65,0.73)	0.62 (0.58,0.65)	0.68 (0.66,0.70)
WC	0.65 (0.63,0.67)	**0.60 (0.58,0.62)**	0.70 (0.67,0.74)	0.62 (0.59,0.65)	0.69 (0.67,0.71)
HC	0.60 (0.58,0.62)	0.56 (0.53,0.58)	0.62 (0.58,0.67)	0.56 (0.53,0.60)	0.61 (0.59,0.63)
WHR	0.63(0.61,0.65)	0.59 (0.57,0.61)	0.69 (0.65,0.73)	0.62 (0.59,0.66)	0.69 (0.67,0.71)
WHtR	**0.66(0.63,0.68)**	**0.60(0.58,0.63)**	**0.72(0.68,0.76)**	**0.63(0.59,0.66)**	**0.70(0.68,0.72)**
BF%	0.65(0.62,0.68)	0.59(0.56,0.62)	0.67(0.62,0.72)	0.62(0.58,0.66)	0.67(0.65,0.70)
FM	0.64(0.62,0.67)	0.58(0.55,0.61)	0.67(0.62,0.71)	0.62(0.58,0.66)	0.68(0.65,0.70)
TF%	0.64(0.61,0.67)	0.59(0.56,0.61)	0.67(0.62,0.71)	0.61(0.57,0.65)	0.67(0.64,0.69)
TFM	0.64(0.61,0.67)	0.58(0.55,0.61)	0.66(0.61,0.71)	0.62(0.58,0.66)	0.67(0.64,0.70)
***Male***					
BMI	0.66(0.63,0.70)	0.63(0.60,0.67)	**0.66(0.61,0.70)**	0.59(0.54,0.63)	0.68(0.65,0.71)
WC	0.65(0.62,0.69)	**0.64(0.62,0.68)**	0.64(0.59,0.68)	0.60(0.55,0.64)	**0.69(0.66,0.72)**
HC	0.61(0.58,0.65)	0.61(0.58,0.65)	0.62(0.57,0.67)	0.53(0.49,0.57)	0.63(0.60,0.66)
WHR	0.64(0.60,0.67)	0.63(0.60,0.66)	0.62(0.58,0.66)	**0.62(0.57,0.66)**	0.67(0.64,0.70)
WHtR	**0.67(0.63,0.70)**	**0.64(0.61,0.68)**	0.64(0.60,0.68)	0.60(0.56,0.65)	**0.69(0.66,0.72)**
BF%	0.61(0.56,0.65)	0.62(0.58,0.66)	0.64(0.58,0.69)	0.56(0.51,0.61)	0.63(0.59,0.67)
FM	0.62(0.58,0.66)	0.63(0.58,0.67)	0.64(0.59,0.69)	0.55(0.50,0.61)	0.64(0.60,0.68)
TF%	0.60(0.56,0.65)	0.62(0.58,0.66)	0.62(0.57,0.68)	0.57(0.51,0.62)	0.63(0.59,0.67)
TFM	0.61(0.57,0.66)	0.62(0.58,0.67)	0.64(0.59,0.69)	0.55(0.50,0.61)	0.64(0.60,0.68)

BMI: body mass index; WC: waist circumference; HC: hip circumference; WHR: waist to hip ratio; WHtR: waist to height ratio; BF: body fat mass; %BF: percentage body fat; TF: trunk fat mass; %TF: percentage trunk fat; MetS: metabolic syndrome.

The bold indicates the highest value of AUC value among the adiposity indices.

We classified the subjects into quartiles according to each adiposity index. The category boundaries were shown in Table S2. Multivariate-adjusted odds ratios (ORs) for metabolic risk factors in the highest (vs. the lowest) quartile of each obesity index are shown in Table 3. In women, the pattern was similar to that found in the ROC analyses. WHtR was consistently the best predictor of all of the studied endpoints. The ORs (95%CI) in the highest (vs. lowest) quartile of WHtR were 3.69 (2.82, 4.83) for hypertension, 2.60 (2.02, 3.36) for dyslipidemia, 7.16 (3.74, 13.73) for hyperuricemia, 2.75 (1.80, 4.20) for diabetes/IFG, and 8.63 (6.26, 11.89) for MetS. In men, BMI showed the best performance in predicting hypertension and hyperuricemia while WHtR was the best predictor for the remaining three endpoints (dyslipidemia, diabetes/IFG, and MetS). The corresponding ORs (95%CI) of the best predictors were 4.54 (3.17, 6.48) for hypertension, 4.03 (2.83, 5.74) for dyslipidemia, 4.28 (2.50, 7.35) for hyperuricemia, 2.92 (1.77, 4.81) for diabetes/IFG, and 6.94 (4.63, 10.40) for MetS. The BIA-derived indices were the second-worst predictors for all of the endpoints, and HC was the worst (Table 3). We compared the predictive potential between the best single adiposity index and a composite factor generated using factor analysis, and found no additive value of the composite factor in the prediction of presence of cardiometabolic disturbance (Table S3).

**Table 3:** Odds ratio (95% CI) of the presence of hypertension, dyslipidaemia, hyperuricemia, and metabolic syndrome for the highest quartile vs. the lowest quartile of adiposity measures*.

	Hypertension	Dyslipidaemia	Hyperuricemia	Diabates/IFG	MetS
***Female***					
BMI	3.92(2.94,5.23)^d^	2.57(1.99,3.31)^d^	4.33(2.53,7.41)^d^	2.42 (1.67,3.51)^d^	5.48 (4.11,7.30)^d^
WC	3.68(2.74,4.94)^d^	2.47(1.91,3.18)^d^	5.61(3.06,10.30)^d^	2.67 (1.76,4.04)^d^	6.81 (5.03,9.23)^d^
HC	2.88 (2.18,3.80)^d^	1.42(1.11,2.82)^b^	2.74(1.70,4.42)^d^	1.75(1.22,2.51)^b^	2.97 (2.27,3.87)^d^
WHR	2.66 (1.97,3.58)^d^	2.53(1.95,3.37)^d^	5.66(3.02,10.60)^d^	2.22(1.46,3.37)^c^	6.15 (4.51,8.39)^d^
WHtR	**4.17 (3.09,5.62)^d^**	**2.60(2.02,3.36)^d^**	**7.16(3.74,13.73)^d^**	**2.75(1.80,4.20)^d^**	**8.63 (6.26,11.89)^d^**
%BF	3.60 (2.62,4.94)^d^	2.23(1.69,2.96)^d^	4.07(2.33,7.12)^d^	2.47(1.66,3.68)^d^	5.07 (3.70,6.96)^d^
BF	3.93(2.86,5.41)^d^	2.09(1.58,2.77)^d^	3.71(2.18,6.35)^d^	2.31(1.57,3.98)^d^	5.08 (3.71, 6.96)^d^
%TF	3.22(2.36,4.39)^d^	2.12(1.60,2.81)^d^	3.87(2.22,6.78)^d^	2.39(1.61,3.55)^d^	4.41 (3.23,6.01)^d^
TF	3.81(2.78,5.25)^d^	2.21(1.67,2.92)^d^	3.55(2.08,6.07)^d^	2.49(1.67,3.72)^d^	5.48 (3.99,7.53)^d^
***Male***					
BMI	**4.90(3.36,7.17)^d^**	3.57(2.52,5.07)^d^	**4.28(2.50,7.35)^d^**	2.34 (1.47,3.74)^c^	5.71(3.91,8.34)^d^
WC	4.00 (2.480,5.83)^d^	3.81(2.68,5.40)^d^	3.99(2.20,7.22)^d^	2.76(1.68,4.52)^c^	6.56(4.44,9.68)^d^
HC	3.17(2.14,4.55)^d^	2.86(2.01,4.05)^d^	3.19(1.86,5.47)^c^	1.34(0.84,2.14)	3.29(2.29,4.73)^d^
WHR	3.69(2.56,5.32)^d^	3.85(2.71,5.46)^d^	3.04(1.72,5.37)^c^	2.58(1.59,4.18)^c^	6.34(4.30,9.37)^d^
WHtR	**4.06(2.78,5.93)^d^**	**4.03(2.83,5.74)^d^**	3.76(2.07,6.83)^d^	**2.92(1.77,4.81)^d^**	**6.94(4.63,10.40)^d^**
%BF	2.42 (1.53,3.81)^c^	3.48(2.22,5.49)^d^	3.37(1.78,6.37)^b^	1.90(1.09,3.31)^a^	3.81(2.37,6.13)^d^
BF	2.64 (1.67,4.18)^c^	3.10(1.98,4.86)^d^	2.99(1.63,5.52)^c^	1.83(1.05,3.21)^a^	3.98(2.50,6.34)^d^
%TF	2.45(1.56.3.87)^c^	3.28(2.09,5.15)^d^	3.19(1.67,6.07)^c^	1.67(0.97,2.88)	3.34(2.10,5.31)^d^
TF	2.47(1.55,3.93)^c^	3.40(2.16,5.35)^d^	4.27(2.15,8.46)^d^	1.77(1.01,3.10)^a^	4.22(2.62,6.79)^d^

* Adjusted for age, smoking status, alcohol intake, and physical activity.

BMI, WC, HC, WHR, WHtR, BF, %BF, TF, %TF, MetS: see Table 2.

a<0.05, b<0.01, c<0.001, d<0.0001.

The bold indicates the highest OR value among the adiposity indices.

## Discussion

We compared both direct and indirect adiposity measurements for screening people at higher risk of hypertension, dyslipidemia, hyperuricemia, diabetes/IFG, and metabolic syndrome in a large sample of middle-aged Chinese. WHtR proved to be the best predictor of most conditions except for hypertension, independent of BMI. BMI was the best screening tool for hypertension in both genders. The measures of BF, %BF, TF, and %TF were substantially weaker predictors and HC was the weakest for all conditions.

### Body mass index

It is accepted that abdominal fat is an important determinant of obesity-associated risk factors, possibly more so than the degree of excess weight as measured by BMI [12]. This is confirmed by our findings, which indicated that BMI was inferior to abdominal measures in predicting dyslipidemia, diabetes/IFG, hyperuricemia (in women), and metabolic syndrome, as demonstrated in previous studies [31], [32], [33]. The high metabolic and inflammatory activity of visceral fat deposits within the abdominal cavity, compared with subcutaneous deposits in other parts of the body, provide a plausible explanation for the superiority of abdominal obesity measures in predicting metabolic risk [34].

Nevertheless, in our data, BMI exhibited the best performance in identifying hyperuricemia in men and was equal to WHtR in predicting hypertension in both sexes. The robust discriminatory power of BMI in terms of hypertension was demonstrated in a previous study of 8940 Chinese adults in which BMI had a closer association with hypertension than measures of central obesity in both women and men [10]. Similar associations have also been observed in studies conducted in other countries, such as Korea [19], Spain [35], and the UK [11]. Increased BMI is associated with increased serum glucose, insulin, aldosterone, and renin levels along with increased sympathetic tone. All of these factors are thought to increase blood pressure by increasing vascular volume or peripheral resistance [36]. However, accumulating evidence points to visceral obesity as the most important risk factor for hypertension [37]. To date, few studies have evaluated the use of different obesity measures for detecting the presence of hyperuricemia. In our data, abdominal obesity was a better predictor of hyperuricemia in women, whereas BMI was a better predictor in men. The reason for this sex difference is unclear, although differences in anatomy, physiology, metabolism, and sex hormones may offer a partial explanation.

### Waist-to-height ratio, waist circumference, and waist-to-hip ratio

Several studies on Asian [3], [4], [5], [6] and Caucasian [32], [38] populations have found WHtR to be superior to WC in identifying cases with cardiovascular risk factors. Meta-analyses provide further confirmation of those findings [33], [39]. In agreement with these findings, our data demonstrated that WHtR had better predictive performance than WC for most conditions, even after controlling for BMI. WHtR may be a better predictor of metabolic risk because it takes height into account. An association between short height and risk of coronary heart disease has been previously reported [40]. Henriksson et al. found that body height had an inverse relation to serum cholesterol and non-HDL cholesterol in middle-aged men, which was independent of BMI and WHR [41]. Thus, it may be important to correct WC for height.

WHR has also been proposed as a good predictor of cardiovascular events [42] and of cardiometabolic risk factors among Tehranian adult men [8]. However, of the three abdominal measures, we found it to have the weakest association with most risk conditions, consistent with previous studies [32]. Differences in subject populations (excluding or including high-risk participants), site of waist measurement (midway between the lower rib and iliac crest, at the narrowest noticeable point, or at the level of the umbilicus), statistical methods (such as adjustment for age and other factors), and the definition of risk factors might collectively lead to these discrepancies. In addition, the validity of WHR has also been questioned as it is more susceptible to measurement error than the other indices. Furthermore, a non-obese and an obese individual could theoretically have the same WHR, as the ratio can remain constant with changes in weight [43]. However, it was noteworthy that WHR's discriminatory performance was superior to that of WC for most conditions after adjusting for BMI, which may be due to the BMI-independent inverse association between HC and cardiometabolic risks (Figure S3).

### Hip circumference

Our results indicate that a larger HC was associated with a lower risk of metabolic disturbances after adjusting for BMI (Figure S3). In line with our findings, previous studies evaluating the separate contributions of waist and hip circumferences to metabolic risk factors have also demonstrated the apparent protective effect of a large HC [44], [45]. Similar associations were also found in the context of future diabetes [46], incidence of first myocardial infarction [42], combined cardiovascular disease, and mortality associated with these diseases [47]. A beneficial adipokine profile together with the long-term entrapment of excess fatty acid might be the main reason for the protective properties of gluteofemoral fat [48].

### Bioelectrical impedance measures of adiposity

Our observations add to the findings of previous studies, which have found no advantage in using BIA measures of body fat rather than simple anthropometric measures in detecting obesity-related complications and metabolic risk factors [11], [19], [21], [49]. However, other studies have shown a higher predictive capability of direct body fat measures for those associations [13], [50], [51], [52]. BIA has been found to be a reliable method for assessing BF and %BF when a protocol is followed that controls for factors that may affect the measurement [53], [54]. Notable exceptions include the assessment of individuals with abnormal hydration and extreme weight ranges (BMI>34 kg/m^2^) [53]. We repeated all of our analyses excluding participants with a BMI ≥28 kg/m^2^ (n = 204 in women and n = 78 in men), and the results were remarkably similar (results not reported). Additionally, the intra-individual variability of BIA in our data ranged from 1.54–2.79%, lower than the error rate reported in previous studies (2–3.5%) [54]. Thus, our findings are robust and not influenced by differential BIA measurements among obese individuals.

### Limitations

First, our study is limited by its cross-sectional design, which precludes causal inferences. Further longitudinal analyses are needed to provide stronger evidence of these associations. Second, people from southern China tend to be shorter and have a lower BMI and WC than those from northern China [55]. Our participants were not randomly selected, which might attenuate their representativeness. Our sample included a low proportion of obese participants, with only 3.0% of women and 2.5% of men having a BMI >29.9 kg/m^2^. The association between obesity and its related risk factors might be underestimated due to the limited variability in the obesity indices. Therefore, caution should be taken in extrapolating our results to the general population and other ethnic groups. Further investigation with a larger sample of obese people is needed.

### Conclusions

Our study favors the use of anthropometric measures of abdominal obesity, especially WHtR, for assessing cardiometabolic disturbances. BMI may be used as an alternative obesity measure for detecting people suffering from hypertension. Further prospective studies are needed before definite conclusions can be made regarding the best predictor of future cardiovascular events.

## Supporting Information

Figure S1 The ROC curves of the best obesity measurement for predicting metabolic risks in female. AUC: area under curve.
(TIF)

Figure S2 The ROC curves of the best obesity measurement for predicting metabolic risks in male. AUC: area under curve.
(DOCX)

Figure S3 Odd ratios (ORs) for the presence of hypertension, dyslipidaemia, hyperuricemia, diabetes/IFG and metabolic syndrome in females and males for the highest quartile vs. the lowest quartile of WC, WHR, WHtR and HC after adjusted by age, smoking status, alcohol intake, physical activity and BMI.
(DOCX)

Table S1 The optimal cut off value, sensitivity, specificity, positive and negative predictive value for the best obesity index in ROC analysis for predicting obesity-associated metabolic risks.
(DOCX)

Table S2 The thresholds for each quartile of obesity indexes in both sexes.
(DOC)

Table S3 The area under the curves (95%CI) and odd ratios (95%CI) of the composite factor and best single adiposity index.
(DOCX)
