# Supplementary material for: Comparison of Various Anthropometric and Body Fat Indices in Identifying Cardiometabolic Disturbances in Chinese Men and Women
Source: PLoS One. 2013 Aug 12;8(8):e70893. doi: 10.1371/journal.pone.0070893 (PMC3741370; doi:10.1371/journal.pone.0070893)
Supplement: Figure S2 — The ROC curves of the best obesity measurement for predicting metabolic risks in male. AUC: area under curve. (DOCX) [file pone.0070893.s002.docx]

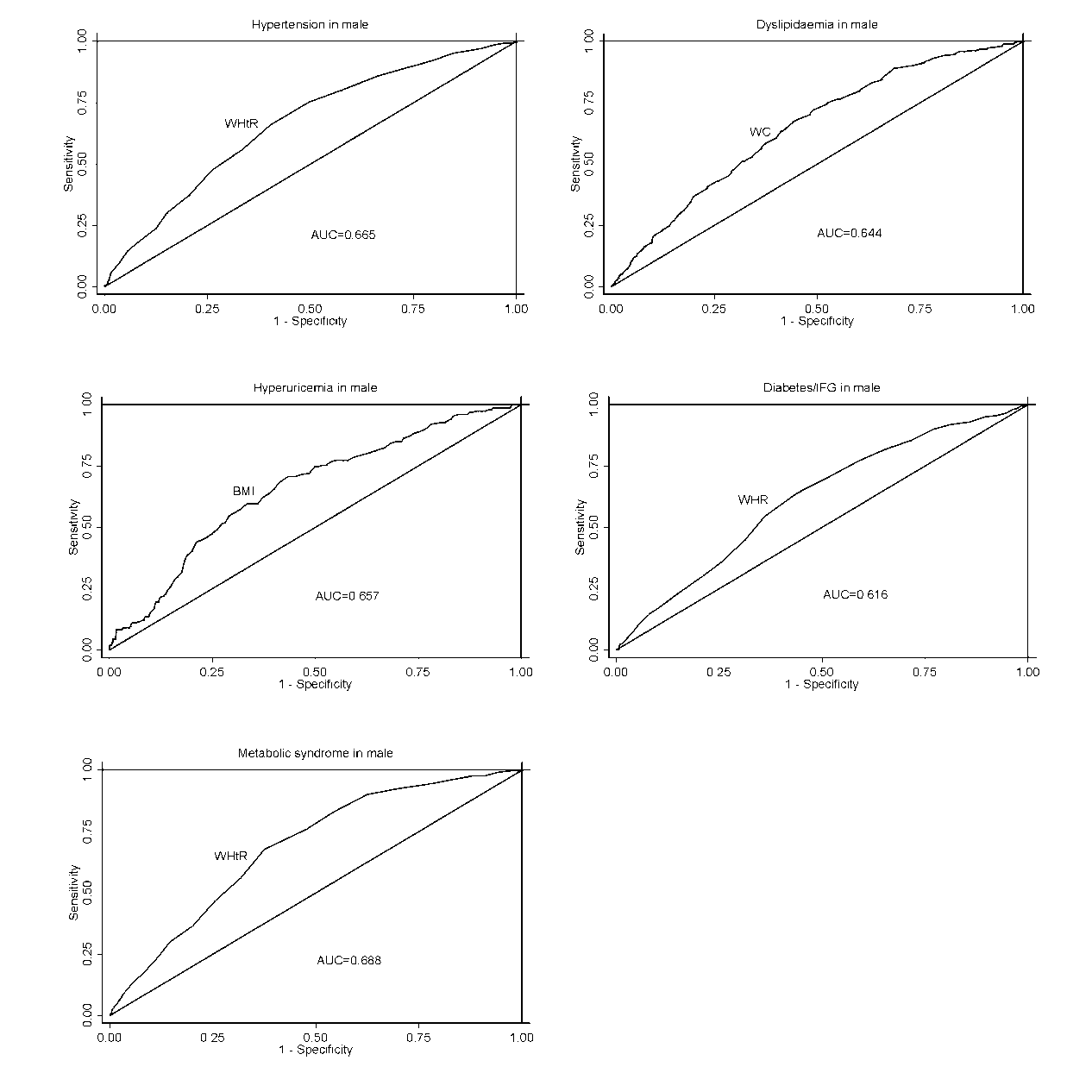


Figure S2. The ROC curves of the best obesity measurement for predicting metabolic risks in male. AUC: area under curve
