# Supplementary material for: Comparison of Various Anthropometric and Body Fat Indices in Identifying Cardiometabolic Disturbances in Chinese Men and Women
Source: PLoS One. 2013 Aug 12;8(8):e70893. doi: 10.1371/journal.pone.0070893 (PMC3741370; doi:10.1371/journal.pone.0070893)
Supplement: Figure S3 — Odd ratios (ORs) for the presence of hypertension, dyslipidaemia, hyperuricemia, diabetes/IFG and metabolic syndrome in females and males for the highest quartile vs. the lowest quartile of WC, WHR, WHtR and HC after adjusted by age, smoking status, alcohol intake, physical activity and BMI. (DOCX) [file pone.0070893.s003.docx]

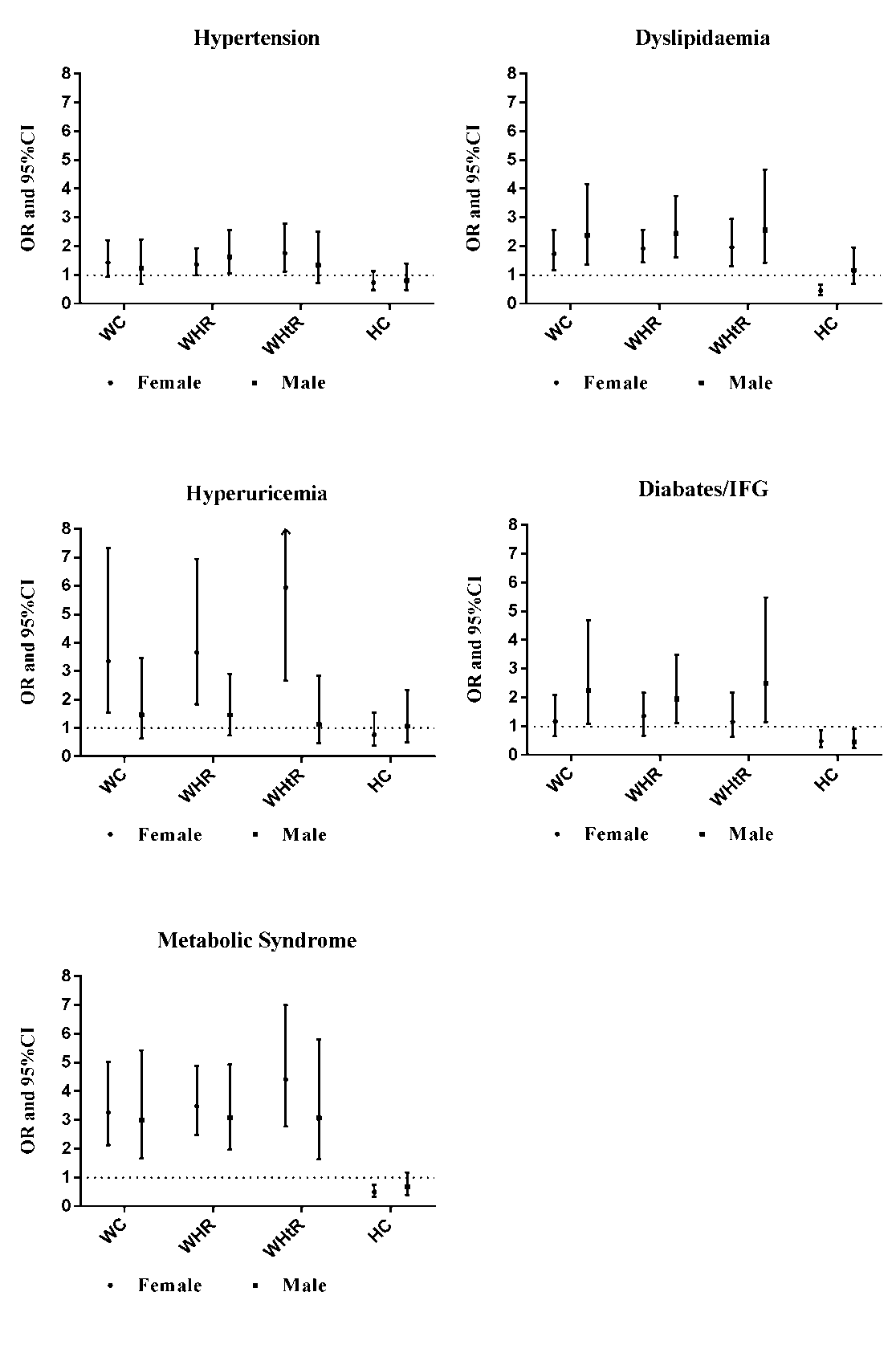


Figure S3. Odd ratios (ORs) for the presence of hypertension, dyslipidaemia, hyperuricemia, diabetes/IFG and metabolic syndrome in females and males for the highest quartile vs. the lowest quartile of WC, WHR, WHtR and HC after adjusted by age, smoking status, alcohol intake, physical activity and BMI.
