# Supplementary material for: Comparison of Various Anthropometric and Body Fat Indices in Identifying Cardiometabolic Disturbances in Chinese Men and Women
Source: PLoS One. 2013 Aug 12;8(8):e70893. doi: 10.1371/journal.pone.0070893 (PMC3741370; doi:10.1371/journal.pone.0070893)
Supplement: Table S1 — The optimal cut off value, sensitivity, specificity, positive and negative predictive value for the best obesity index in ROC analysis for predicting obesity-associated metabolic risks. (DOCX) [file pone.0070893.s004.docx]

Table S1. The optimal cut off value, sensitivity, specificity, positive and negative predictive value for the best obesity index in ROC analysis for predicting obesity-associated metabolic risks.

|  | Best Index* | Optimal cut-off | Sensitivity  (%) | Specificity  (%) | Positive predictive value (%) | Negative predictive value (%) |
| --- | --- | --- | --- | --- | --- | --- |
| Female |  |  |  |  |  |  |
| Hypertension | WHtR | 0.52 | 66.0 | 56.4 | 38.6 | 80.0 |
| Dyslipidaemia | WHtR | 0.54 | 45.4 | 69.9 | 57.8 | 58.5 |
| Hyperuricemia | WHtR | 0.52 | 81.2 | 52.1 | 12.2 | 97.1 |
| Diabates/IFG | WHtR | 0.55 | 47.0 | 71.6 | 18.5 | 90.8 |
| MetS | WHtR | 0.51 | 75.9 | 52.7 | 44.9 | 81.2 |
| Male |  |  |  |  |  |  |
| Hypertension | WHtR | 0.51 | 75.2 | 50.4 | 46.7 | 77.9 |
| Dyslipidaemia | WC | 84.8 | 67.1 | 56.1 | 61.5 | 62.0 |
| Hyperuricemia | BMI | 23.8 | 70.8 | 56.6 | 23.0 | 91.4 |
| Diabates/IFG | WHR | 0.92 | 64.0 | 56.0 | 21.1 | 89.4 |
| MetS | WHtR | 0.52 | 67.8 | 62.5 | 52.5 | 76.0 |

BMI, WC, HC, WHR, WHtR, BF, %BF, TF, %TF: see supplemental Table 1; MetS: metabolic syndrome.

*: with the highest value of area under the curves in ROC analysis.
