# Supplementary material for: Comparison of Various Anthropometric and Body Fat Indices in Identifying Cardiometabolic Disturbances in Chinese Men and Women
Source: PLoS One. 2013 Aug 12;8(8):e70893. doi: 10.1371/journal.pone.0070893 (PMC3741370; doi:10.1371/journal.pone.0070893)
Supplement: Table S2 — The thresholds for each quartile of obesity indexes in both sexes. (DOC) [file pone.0070893.s005.doc]

Table S2. The thresholds for each quartile of obesity indexes in both sexes.

|  | Quartile1 | Quartile2 | Quartile3 | Quartile4 |
| --- | --- | --- | --- | --- |
| **Female** |  |  |  |  |
| BMI | ≤20.9 | 21.0-22.8 | 22.9-25 | >25 |
| WC | ≤74.4 | 74.5-80.4 | 80.5-86.5 | >86.5 |
| HC | ≤89 | 89.1-92.9 | 93.0-97.0 | >97.0 |
| WHR | ≤0.82 | 0.82-0.86 | 0.86-0.91 | >0.91 |
| WHtR | ≤0.48 | 0.48-0.52 | 0.52-0.55 | >0.55 |
| BF% | ≤26.8 | 26.9-31.0 | 31.1-35.0 | >35.0 |
| FM | ≤13.4 | 13.5-17.1 | 17.2-21.5 | >21.5 |
| TF% | ≤24.8 | 24.9-29.9 | 30.0-34.9 | >34.9 |
| TFM | ≤6.7 | 6.8-9.1 | 9.2-11.6 | >11.7 |
| **Male** |  |  |  |  |
| BMI | ≤21.7 | 21.7-23.6 | 23.6-25.5 | >25.5 |
| WC | ≤80.5 | 80.5-85.7 | 86.0-91.0 | >91.0 |
| HC | ≤90.4 | 90.5-93.8 | 94-97.5 | >97.5 |
| WHR | ≤0.87 | 0.87-0.91 | 0.91-0.95 | >0.95 |
| WHtR | ≤0.48 | 0.48-0.51 | 0.51-0.55 | >0.55 |
| BF% | ≤16.6 | 16.7-20.0 | 20.1-23.2 | >23.2 |
| FM | ≤10.1 | 10.2-13.1 | 13.2-16.5 | >16.5 |
| TF% | ≤16.2 | 16.2-20.9 | 21.0-24.8 | >24.8 |
| TFM | ≤5.3 | 5.4-7.3 | 7.4-9.3 | >9.3 |

BMI: body mass index; WC: waist circumference; HC: hip circumference; WHR: waist to hip ratio; WHtR: waist to height ratio; BF: body fat mass; %BF: percentage body fat; TF: trunk fat mass; %TF: percentage trunk fat; MetS: metabolic syndrome.
