# Supplementary material for: Comparison of Various Anthropometric and Body Fat Indices in Identifying Cardiometabolic Disturbances in Chinese Men and Women
Source: PLoS One. 2013 Aug 12;8(8):e70893. doi: 10.1371/journal.pone.0070893 (PMC3741370; doi:10.1371/journal.pone.0070893)
Supplement: Table S3 — The area under the curves (95%CI) and odd ratios (95%CI) of the composite factor and best single adiposity index. (DOCX) [file pone.0070893.s006.docx]

Table S3. The area under the curves (95%CI) and odd ratios (95%CI) of the composite factor and best single adiposity index

|  | Hypertension | Dyslipidaemia | Hyperuricemia | Diabates/IFG | MetS |
| --- | --- | --- | --- | --- | --- |
| AUC (95% CI) | | | | | |
| Female |  |  |  |  |  |
| Composite ^*^ | 0.66 (0.64, 0.68) | 0.60(0.58, 0.62) | 0.71 (0.67, 0.75) | 0.63(0.59, 0.66) | 0.70(0.68, 0.72) |
| Best ^#^ | 0.66(0.63,0.68) | 0.60(0.58,0.63) | 0.72(0.68,0.76) | 0.63(0.59,0.66) | 0.70(0.68,0.72) |
| Male |  |  |  |  |  |
| Composite | 0.66(0.63, 0.69) | 0.65(0.62, 0.68) | 0.65(0.60, 0.69) | 0.60(0.56, 0.64) | 0.69(0.66, 0.72) |
| Best | 0.67(0.63,0.70) | 0.64(0.61,0.68) | 0.66(0.61,0.70) | 0.62(0.57,0.66) | 0.69(0.66,0.72) |
| OR (95% CI) | | | | | |
| Female |  |  |  |  |  |
| Composite | 3.95(2.94, 5.29) | 2.54(1.97, 3.27) | 6.21(3.39, 11.37) | 2.28(1.55, 3.35) | 6.64(4.92, 8.98) |
| Best | 4.17 (3.09,5.62) | 2.60(2.02,3.36) | 7.16(3.74,13.73) | 2.75(1.80,4.20) | 8.63 (6.26,11.89) |
| Male |  |  |  |  |  |
| Composite | 4.19(2.86, 6.12) | 3.85(2.71, 5.46) | 3.66(2.07, 6.47) | 3.05(1.87, 4.98) | 6.74(4.56, 9.97) |
| Best | 4.90(3.36,7.17) | 4.03(2.83,5.74) | 4.28(2.50,7.35) | 2.92(1.77,4.81) | 6.94(4.63,10.40) |

^*^: Composite factor (Female) = 0.919 BMI + 0.786 HC + 0.985 WC + 0.752 WHR + 0.969 WHtR; Composite factor (Male) = 0.930 BMI + 0.810 HC + 0.986 WC + 0.817 WHR + 0.969 WHtR. The composite factor was derived from factor analysis.

^#^: Best single adiposity index: see Table 2 and Table 3.

BMI, WC, WHR, WHtR, MetS: see supplemental Table 2

AUC, area under the curves; OR, odd ratio
